# Pathobiology and Chemoprevention of Bladder Cancer

**DOI:** 10.1155/2011/528353

**Published:** 2011-09-15

**Authors:** Takuji Tanaka, Katsuhito Miyazawa, Tetsuya Tsukamoto, Toshiya Kuno, Koji Suzuki

**Affiliations:** ^1^The Tohkai Cytopathology Institute: Cancer Research and Prevention (TCI-CaRP), 5-1-2 Minami-Uzura, Gifu 500-8285, Japan; ^2^Department of Oncologic Pathology, Kanazawa Medical University, 1-1 Daigaku, Uchinada, Ishikawa 920-0293, Japan; ^3^Department of Urology, Kanazawa Medical University, 1-1 Daigaku, Uchinada, Ishikawa 920-0293, Japan; ^4^Department of Pathology, Fujita Health University School of Medicine, 1-98 Dengakugakubo, Kutsukake-cho, Toyoake, Aichi 470-1192, Japan; ^5^Department of Tumor Pathology, Gifu University Graduate School of Medicine, 1-1 Yanagido, Gifu City, Gifu 501-1194, Japan

## Abstract

Our understanding of the pathogenesis of bladder cancer has improved considerably over the past decade. Translating these novel pathobiological discoveries into therapies, prevention, or strategies to manage patients who are suspected to have or who have been diagnosed with bladder cancer is the ultimate goal. In particular, the chemoprevention of bladder cancer development is important, since urothelial cancer frequently recurs, even if the primary cancer is completely removed. The numerous alterations of both oncogenes and tumor suppressor genes that have been implicated in bladder carcinogenesis represent novel targets for therapy and prevention. In addition, knowledge about these genetic alterations will help provide a better understanding of the biological significance of preneoplastic lesions of bladder cancer. Animal models for investigating bladder cancer development and prevention can also be developed based on these alterations. This paper summarizes the results of recent preclinical and clinical chemoprevention studies and discusses screening for bladder cancer.

## 1. Introduction

**Figure 1:**
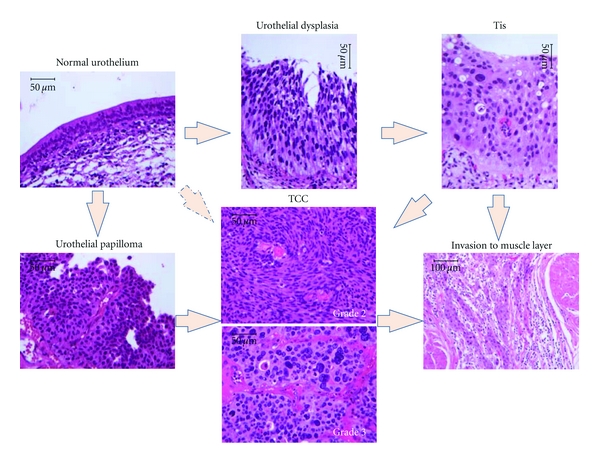
Natural history of bladder cancer (transitional cell carcinoma). Tis, transitional cell carcinoma in situ; and TCC, transitional cell carcinoma.

**Figure 2:**
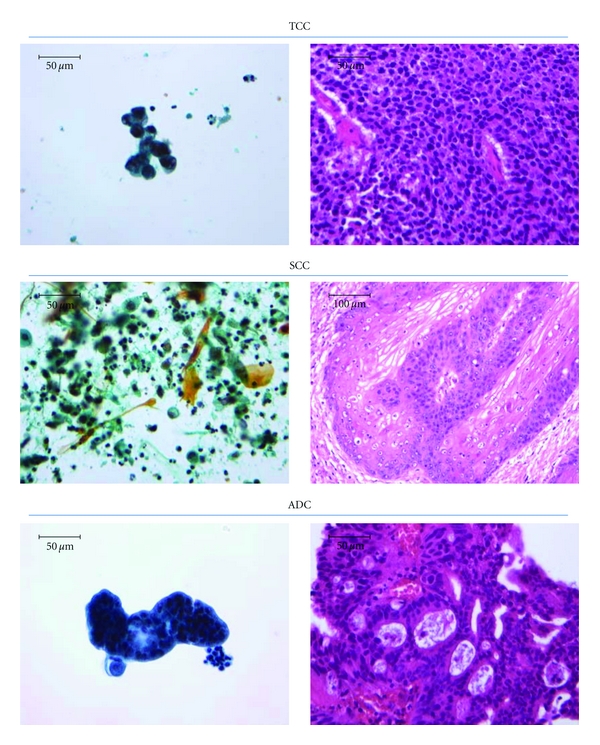
Three main histological types of bladder cancer and their urinary cytology. TCC, transitional cell carcinoma; SCC, squamous cell carcinoma; and ADC, adenocarcinoma.

There has recently been an increasing incidence of and significant mortality rates attributed to bladder cancer. Fortunately, our understanding of the pathobiology of this malignancy has improved considerably over the past decade. Translating these novel pathobiological discoveries into therapies or strategies to manage patients who are suspected to have or who have been previously diagnosed with bladder cancer is the ultimate goal. Of the three main histological variants of epithelial malignancies arising from the urothelium of the urinary bladder, transitional cell carcinoma (TCC, Figure 1) is the most prevalent in Japan, North America, and other developed countries, while squamous cell carcinoma (Figure 2) and adenocarcinoma (Figure 2) are diagnosed less frequently. 

In this paper, the reported alterations of both oncogenes and tumor suppressor genes in bladder cancer will be outlined and described in the context of possible novel therapies targeting these alterations. Several investigators have hypothesized that certain chromosomal abnormalities and mutations play definite roles in bladder cancer development, while other alterations correlate with tumor progression. This paper will also describe the molecular basis of known malignant phenotypes. These findings will provide insight into the biological and clinical significance of various preneoplastic lesions. In addition, we will summarize the reports in the areas of chemoprevention and screening for bladder cancer. Emphasis will be placed on how the novel biological findings have impacted the development of tests to screen for bladder cancer and on the pathogenesis and chemoprevention of bladder cancer. Finally, animal models are useful for investigating cancer development and prevention, so we will discuss the use of animal models for studying bladder carcinogenesis and bladder cancer prevention and treatment.

## 2. Epidemiology of Bladder Cancer

An estimated 386.300 new cases and 150.200 deaths from bladder cancer were diagnosed worldwide in 2008 [1]. The majority of bladder cancer occurs in males, and there is a 14-fold variation in incidence internationally. The highest incidence rates are found in the countries of Europe, North America, and Northern Africa. Egyptian males have the highest mortality rate (16.3 per 100.000), which is twice as high as the highest rates in Europe (8.3 in Spain and 8.0 in Poland) and more than 4 times higher than that in the United States (3.7) [2]. The countries of Melanesia and Middle Africa have the lowest reported rates. Smoking and occupational exposures are the major risk factors in Western countries, whereas chronic infection with *Schistosoma haematobium* in developing countries, particularly in Africa and the Middle East, accounts for about 50% of the total burden [3]. The majority of bladder cancers associated with schistosomiasis are histologically squamous cell carcinoma, while those associated with smoking are TCC [4].

## 3. Histopathology and Natural History of Bladder Cancer

TCC can be classified into two groups with distinct behavioral and molecular profiles: low-grade cancers (always papillary and usually superficial) and high-grade cancers (either papillary or nonpapillary, and often invasive) [5–9]. Superficial bladder cancers, such as stages Ta (superficial), Tis (in situ, Figure 1), and T1 (early invasive) account for 75% to 85% of neoplasms at clinical presentation, while the remaining 15% to 25% are invasive (T2, T3, and T4) or have metastasized at the time of diagnosis [10]. More than 70% of all patients treated for superficial bladder cancer will subsequently develop one or more recurrent tumors, and about one-third of these patients will progress to cancer that invades the surrounding muscle [11]. 

Several different preneoplastic lesions for bladder cancer have been postulated. Certain hyperplastic changes or everted papillomas (Figure 1) are considered to be significant preexisting conditions (i.e., precursors) for the development of overt papillary transitional cell carcinomas of low grade. The other precursor lesions described within the current nomenclature are related to the flat Tis pathway of tumorigenesis, including the changes known as intra-urothelial neoplasms [12]. This latter group includes simple hyperplasia, atypical urothelial hyperplasia, and dysplasia (Figure 1) or marked atypia. Some authors have used the term Tis grades 1, 2, and 3 for mild, moderate, and severe dysplasia, respectively [13]. Several studies have revealed the importance of diagnosing concomitant dysplasia, as it increases the risk of tumor progression for patients affected with superficial bladder cancers [14, 15]. Finally, squamous metaplasia followed by dysplastic changes is generally accepted as being a premalignant lesion associated with squamous cell carcinoma in bilharzial-related bladder cancer (BBC). Regardless of the terminology, it is becoming increasingly evident that the morphological changes and the clinical manifestations of bladder cancer are preceded by molecular and biochemical alterations. 

The identification of the multiple and complex chromosomal alterations in bladder TCC has led to the development of the “clonal” theory of bladder cancer pathogenesis, which postulates that multifocal and recurrent tumors evolve from a single transformed cell, from which all progenies derive several identical genetic mutations [16]. The more traditional “field cancerization” theory assumes an overall change in the urothelium, with many transformed cells evolving independently into tumors, and therefore being genetically unrelated [17]. The evidence for both theories is compelling [18]. In reality, these theories are equally valid and both processes can occur simultaneously in the same patient [19]. 

In rats, urothelial carcinogenesis involves a sequence of morphologic changes beginning as simple hyperplasia. It then progresses to nodular and papillary hyperplasia. These progress to papillomas and can eventually progress to higher-grade, noninvasive carcinomas and ultimately to invasive neoplasms (Figure 1) [20]. Many exophytic tumors induced in rats are polypoid, often pedunculated and with an inverted papillary growth pattern [20]. Nodular hyperplasia, in mice, is considerably more common than papillary proliferation, and nodular hyperplasia frequently occurs with a complete absence of papillary hyperplasia [21]. Therefore, the rat model more strongly resembles papillary neoplasms, while the mouse model resembles flat urothelial lesions, both of which have been identified in humans [22].

## 4. Risk Factors for Bladder Cancer

Aside from age, gender, and race, exposure to numerous environmental agents and chemicals has been closely correlated with the risk of developing bladder cancer. Most environmental bladder carcinogens are aromatic amines. A Chinese herbal mixture containing *Stephania tetrandra* and *Magnolia officinalis* that was imported into Belgium as a popular weight-reduction aid primarily used by women became responsible for an epidemic of interstitial nephropathy, presumably from contamination with *Aristolochia fangchi,* which had been substituted for *S. tetrandra* [23]. Subsequently, patients with Chinese herb nephropathy have been reported to be at much higher risk for developing urothelial carcinoma, primarily of the upper urinary tract, but also of the bladder [24]. A major mechanism causing this condition appears to be the development of aristolochic acid-related DNA adducts in the urothelium of both the upper urinary tract and bladder. Public health measures for restricting the distribution of these herbs are now in place in many countries [25]. 

### 4.1. Occupational Exposure

The aniline dyes used to color fabrics are urothelial carcinogens [1]. Other chemicals that have been shown to be carcinogens for bladder cancer include 2-naphthylamine, 4-aminobiphenyl, 4-nitrobiphenyl, 4-4-diaminobiphenyl (benzidine), and 2-amino-1-naphthol [26]; combustion gases and soot from coal; possibly chlorinated aliphatic hydrocarbons [26]; certain aldehydes such as acrolein used in chemical dyes and in the rubber and textile industries [5]. Other potential sources of carcinogenic compounds are dietary nitrites and nitrates that are acted upon by intestinal bacterial flora [27, 28] and contaminants of ingested herbal remedies such as aristolochic acid [24]. Occupations reported to be associated with an increased risk of bladder cancer include autoworkers, painters, truck drivers, drill press operators, leather workers, metal workers, and machinery operators, as well as those occupations that involve organic chemicals, such as dry cleaners, paper manufacturing, rope and twine makers, dental technicians, barbers or beauticians, physicians, workers in apparel manufacturing, and plumbers [29, 30].

### 4.2. Cigarette Smoking

Active cigarette smokers have a two- to fourfold higher incidence of bladder cancer than do people who have never smoked [31]. The specific chemical carcinogen responsible for bladder cancer in cigarette smoke has not been identified. Nitrosamines, 2-naphthylamine, and 4-aminobiphenyl are known to be present, and increased urinary tryptophan metabolites have also been demonstrated in cigarette smokers [32]. Two-thirds of bladder cancer cases may be related to cigarette smoking and the risk, in both sexes, correlates well with the number of cigarettes smoked, the duration of smoking, and the degree of smoke inhalation [33]. Smoking cessation significantly reduces the bladder cancer risk, however, even after 20–25 years, it never reaches the baseline risk level of nonsmokers. Regardless of these factors, from a clinical standpoint, it is important to realize that not only does smoking increase the risk for developing bladder cancer, but also that failure to quit smoking once a diagnosis is made predicts a worse outcome, even in those initially diagnosed with noninvasive cancers [34]. Other forms of tobacco use are associated with only a slightly higher risk for bladder cancer than the general population [31].

### 4.3. Infection

Urinary tract infection and chronic irritation are associated with an increased risk of squamous cell carcinoma of the bladder [35]. Two to 10% of paraplegics with long-term indwelling catheters develop bladder cancer, 80% of which are squamous cell carcinomas. Managing these patients without chronic indwelling catheters decreased the incidence of bladder cancer and the preponderance of squamous cell carcinomas [36]. Unfortunately, despite these favorable trends, well over half of these patients have invasive cancers at diagnosis, a figure that is more than double that found in the general population without spinal cord injuries [37]. Despite this high risk, periodic screening via cystoscopy and/or cytology (Figure 2) for patients with long-term indwelling catheters (in the absence of gross hematuria) is not strongly supported [38]. On the other hand, this clearly is a prime population for secondary preventive efforts. 

Bladder infection by *Schistosoma haematobium*, especially in endemic areas like Egypt, appears to be causally related to the development of both squamous cell carcinoma and less frequently, transitional cell carcinomas. The mechanisms of carcinogenesis are not yet understood but may involve the formation of nitrite and *N*-nitroso compounds in the bladder [39], presumably from parasitic (or microbial, transmitted with the parasite) metabolism of normal urinary constituents [40]. 

The role of exposure to the human papillomavirus (HPV) in bladder cancer has been evaluated by several groups, with widely divergent findings. Previous reports have indicated that as few as 2% [41] to as high as 35% of human bladder cancers are contaminated by HPV DNA [42]. Griffiths and Mellon [43] concluded that this virus was more likely to play a role in transitional cell tumorigenesis in immunocompromised hosts, rather than in cancers arising in immunologically competent individuals. The role of other viral agents in the etiology of transitional cell cancer has been investigated but not yet fully elucidated [44].

### 4.4. Radiation and Other Therapies

Female patients treated with radiotherapy for carcinoma of the uterine cervix or ovary have a twofold to fourfold increased risk of developing bladder cancer compared with women only undergoing surgery [45]. The incidence rises further if chemotherapy was also administered (with or without cyclophosphamide) or even if chemotherapy was used alone. The risks in all groups continued to rise for 10 years after treatment [45]. These tumors are characteristically high grade and locally advanced at the time of diagnosis [45]. There is growing evidence that an increased risk also occurs in males treated by external beam irradiation for prostate cancer [46]. 

Similarly, a risk due to exposure to irradiation as seen in the Chernobyl nuclear reactor accident is still present. Patients undergoing evaluation for treatment due to urinary retention or chronic abacterial cystitis from regions that were considered to be contaminated excreted far higher amounts of ^137^Cs in their urine more than 15 years after the nuclear accident than those residing in uncontaminated areas and had significantly more proliferative and dysplastic lesions on mucosal biopsies than nonexposed individuals. In addition, they had a 52% incidence of histological carcinoma in situ and a 6.4% incidence of urothelial carcinomas compared to 0% in patients from uncontaminated areas [47]. 

The patients treated with cyclophosphamide and exposed to its urinary metabolite, acrolein [48], have up to a ninefold increased risk of developing bladder cancer although the specific relationship has not been formally demonstrated in case-control epidemiological studies [49]. Most of these high-grade and muscle-infiltrating tumors develop 6–13 years after exposure and occur in patients younger than those with sporadic urothelial cancer and have an equal incidence in both sexes [50]. Studies suggest that the uroprotectant, mesna (2-mercaptoethanesulfonic acid), may reduce the risk of bladder cancer [51]. Some authors suggest aggressive therapy upon diagnosis (e.g., cystectomy), even when the tumor is still noninvasive, because of the unusually high rate of progression experienced by patients in whom cystectomy is withheld [52]. 

Black-foot disease is endemic in South Taiwan, and it is usually associated with vascular and cardiac disease and with the development of numerous malignancies, including transitional cell cancer of the bladder [53]. This condition appears to be related to ingestion of large quantities of arsenic in artesian well water. Similar endemic pockets of bladder cancer are found in other regions with high arsenic concentrations in drinking water [54]. In a nested case-control study, Liou et al. [53] demonstrated that specific cytogenetic abnormalities, including chromosome-type breaks, gaps, exchanges, and other aberrations, were more frequent in peripheral blood cells and urothelial cells of exposed patients who ultimately developed cancer over a 4-year period of observation compared with exposed individuals who did not. Regardless of the mechanism(s) of oncogenesis in this entity, the incidence appears to be declining with effective public health measures focused on avoiding contaminated water. 

Renal transplant recipients [55] and individuals having a chronically low amount of fluid ingestion [56] are also at an increased risk for developing bladder cancer. Transplant recipients, presumably because of prolonged immunosuppression [54], are known to be at a higher risk for developing numerous tumors. Similarly, if certain chemicals are responsible for initiating mutational events, prolonged exposure to higher concentrations of them is likely to be more mutagenic/carcinogenic than exposure to lower concentrations.

### 4.5. Hereditary Factors

Strong epidemiological evidence does not exist for a hereditary cause of most cases of bladder cancer. Perhaps the most compelling evidence in this regard comes from the work of Klemeney and Schoenberg [57], who studied the records of more than 12,000 relatives of 190 patients diagnosed with transitional cell cancer in Iceland between 1983 and 1992. They found that while the risk of developing transitional cell carcinoma was slightly elevated in relatives (observed-to-expected odds ratio of 1.24, 95% confidence interval of 0.9 to 1.67), this ratio was greater among second- and third- than first-degree relatives. This argues strongly against a straightforward genetic mechanism being responsible. Familial clusters of bladder cancer have been reported [57]. However, most of the authors did not report whether this increased risk in the affected families was observed only in relatives who were smokers (or having exposure to other putative carcinogens). This is important because Kantor and associates [35] indicated that the increased familial risk was primarily in relatives who smoked. The correlations between familial predisposition, possible exposure, and some of the genotypic/phenotypic analyses of enzymes responsible for the activation or inactivation of putative bladder cancer carcinogens found in cigarette smoke are therefore required to permit the identification of at-risk individuals who may be the best subjects for interventions such as avoidance, prevention, and early detection strategies.

## 5. Screening for Bladder Cancer

As the natural history of bladder cancer is characterized by multiple tumor recurrences and a risk of disease progression, it is imperative that screening tests of high sensitivity and specificity are available for evaluating patients at increased risk of developing bladder cancer and for patients previously treated for superficial bladder cancer. Cystoscopy is the gold standard against which all other tests are currently compared. However, this test is both invasive and costly. As a result, considerable emphasis has therefore been placed on developing noninvasive tests for screening for bladder cancer. Currently, the following noninvasive tests are either being used in clinical practice or undergoing evaluation as screening tests for bladder cancer: urine cytology (Figure 2), bladder tumor antigen (BTA) (TRAK assay, Bard Diagnostic Sciences, Redmond, Wash), nuclear matrix protein (NMP) test (Matritech, Newton, Mass), fibrin degradation product (FDP) assay (Perlmmune, Rockville, Md), hyaluronic-acid (HA-) hyaluronidase (HAase) urine test, and urine detection of surviving [58]. With regard to gold standard for a non-invasive screening test for bladder cancer, urine cytology is inadequate due to its low sensitivity and subjective diagnostic criteria. This has led to the development of the other screening tests, and each of these tests should be compared to the results of urine cytology to determine whether it might offer better diagnostic accuracy and sensitivity. The overall sensitivity of urine cytology has been reported to be between 40 and 60%, with higher sensitivities being reported from studies with a higher proportion of patients diagnosed with high-grade tumors [12, 59, 60]. Although urine cytology has a low sensitivity, it has consistently demonstrated excellent specificity. We previously proposed the use of the number of silver-stained nucleolar organizer regions of urothelial cells in urine as a biomarker to detect urothelial malignancy [61]. Determining the lactate dehydrogenase isoenzymes in urinary cytology has also shown good diagnostic results [62].

## 6. Molecular Pathway of Bladder Carcinogenesis

### 6.1. Growth Signaling

Cancer cells have less dependence on exogenous growth stimulation, generating many of their growth signals in an autocrine fashion or by overexpressing growth signal receptors to become hyperresponsive to normal tissue levels of growth factors [63]. Growth factor receptors tend to have tyrosine kinase activity in their cytoplasmic domains and are thus able to activate several intracellular signaling pathways [63]. Downstream of the growth factors, the mitogen-activated protein kinase (MAPK) pathway appears to be central, and mutations in its components enable mitogenic signals to be propagated in the absence of ongoing upstream stimulation [63].

The epidermal growth factor receptor (EGF-R) is a 175-kDa transmembrane glycoprotein which is activated by the binding of epidermal growth factor (EGF) and transforming growth factor (TGF)-*α* [64] to its external domain, as well as by betacellulin [64], epiregulin [65], amphiregulin [64], and heparin-binding EGF-like factor [64], resulting in proliferation, transformation, and division [66]. In bladder TCCs, EGF-R expression is associated with a high tumor grade [67] and stage [68], and rapid tumor proliferation [69], and EGF-R mRNA can be used to detect circulating neoplastic cells in patients' blood [70]. TGF-*α* is considered to be the more important ligand for EGF-R in bladder tumors [71], and its expression correlates strongly with death from bladder cancer [72]. *In vitro*, EGF-R overexpression increases bladder cancer cell motility [20], and EGF can stimulate cancer cell growth and proliferation [73], as well as invasion [74].

Hepatocyte growth factor (HGF) stimulates cell motility, morphogenesis, and angiogenesis *in vitro* [75], and its receptor (encoded by the c-met proto-oncogene) has tyrosine kinase activity [76]. The serum and tissue levels of HGF are significantly higher in patients with muscle-invasive tumors than in those with Ta/T1 bladder cancer, and both the urinary and serum levels may be useful predictors of the extent of disease and patient survival [77]. Although HGF induces cell migration, invasion, and tumorigenicity in malignant cell lines *in vitro*, it only stimulates proliferation in nontumorigenic cell lines [78]. It has been proposed that HGF is released *in vivo* by stromal cells and has differential effects on transformed and nontransformed urothelium [76]. 

Fibroblast growth factors (FGFs) make up a family of 22 human polypeptide growth factors that have important and diverse roles in embryonic development, which in adults can contribute to the pathogenesis of cancer [79]. FGF-2 (basic FGF) expression is elevated in high-stage bladder tumors and correlates with early local recurrence [80]. FGF-1 (acidic FGF) levels in the urine significantly correlate with the stage of disease [81], and *in vitro*, FGF-1 stimulation converts cells from a noninvasive to a metastatic phenotype [82]. FGF-2 also increases the invasive potential of bladder cancer cell lines [83]. FGF receptor-3 (FGF-R3) and TP53 mutations have been recognized as the key genetic pathways in the carcinogenesis of TCC; mutation of the former, being the most frequently mutated oncogene, is strongly associated with a low tumor grade, an early stage, and a low recurrence rate, and mutation of the latter is associated with a higher tumor grade, more advanced stage, and more frequent tumor recurrence [84].

Integrins are transmembrane receptors with diverse cell adhesion and signaling functions, affecting cell growth and differentiation [85] and determining how cells interact with the extracellular matrix [85]. The normal urothelium expresses integrins *α*2, *α*3, *β*1, and *β*4, but not a1, a4, or *α*5 [86]. There is a loss of *α*2 expression as normal urothelial tissue progresses to invasive TCC [86]. Integrin *α*6-*β*4 is expressed by the basal layer of the normal urothelium and low-stage TCCs, whereas invasive bladder cancers show loss of *α*6-*β*4, and its loss can serve as a prognostic factor [87]. However, the specific roles of integrin signaling in bladder carcinogenesis remain to be fully elucidated. 

Many receptor tyrosine kinases and integrins activate a sequential intracellular protein kinase cascade, termed the MAPK module [39]. The role of the MAPK pathway in the transduction of signals from membrane-bound receptors (e.g., EGF-R and integrins) means that its specific therapeutic manipulation is an attractive approach to cancer therapy, and work in this area is already underway [88].

### 6.2. Antigrowth Signaling

In addition to growth stimulation, antiproliferative signals also operate to maintain quiescence and tissue homeostasis [63]. These antigrowth signals are similarly received by cell-surface transmembrane receptors linked to intracellular signaling pathways [63]. Most anti-proliferative signals use the retinoblastoma protein (pRB) and the related p107 and p130 proteins [89]. Cyclin-dependent kinases (CDKs) stimulate cell proliferation, while CDK inhibitors block cell proliferation [90] and inhibit progression from the G2 to the M phase of the cell cycle [91]. Many CDK inhibitors have been identified: p2l^WAF1/Cip1^, p27^Kip1^, p57^Kip2^ (the Cip/Kip family), p15/INK4b, p16/INK4a, p18/INK4c, and p19/INK4d (the INK4 family) [90]. 

pRB expression is frequently lost in bladder tumors and is significantly correlated with high-grade cancer [92] and poor survival [93]. In addition, abnormal expression of both pRB and p53 is significantly associated with tumor proliferation, muscle invasion, high grade, and higher recurrence and progression rates than in patients with alterations in only one of these proteins or no alterations in either [94]. The p53 protein will be discussed in more detail later. Deletions and mutations of the INK4 genes also occur frequently in Ta/T1 bladder cancer, although only deletions affecting both p16 and p19, deregulating both the pRB and p53 pathways, correlate with a worse prognosis [95]. Reduced expression of p27 is also significantly associated with muscle invasion [96]. Therefore, both the pRB and p53 pathways are usually inactivated in bladder cancer that invades the muscle layer [97], and such mutations and inactivations may act synergistically to promote tumor progression [94]. 

The soluble signaling molecule TGF*β* is a well-characterized antigrowth signal that induces the synthesis of p15 and prevents cell-cycle progression [63]. Reduced expression of the TGF*β*1 gene in bladder tumors is significantly associated with high-grade [98] and advanced disease [99]. In addition, loss of expression of the TGF-*β* receptors is significantly associated with a higher tumor grade and stage, the presence of lymph node metastases, progression, and reduced survival [96, 99].

### 6.3. Apoptosis Pathway

The prolonged survival of cells harboring an abnormal genome and the expansion of the resulting tumor rely not only on the rate of cell proliferation but also on the rate of cell loss [63], and an acquired resistance to apoptosis (programmed cell death) is a hallmark of most, or possibly all, cancers [63]. The apoptotic pathway comprises a complex arrangement of sensors, effectors, and regulators [63]. Kelly et al. [100] reviewed this area specifically for bladder cancer. Survival factors include IGF-1 and IGF-2 binding to their receptor IGF-1R [101] and interleukin-3 binding to its receptor interleukin-3R [102]. Death signals include the Fas ligand, which binds to the Fas receptor, tumor necrosis factor (TNF)-*α*, which binds to TNF-R1, and the CD40 ligand binding to CD40 [103]. The apoptotic pathway is also activated in response to DNA damage, signaling imbalances due to oncogene activation, survival factor insufficiency, or hypoxia [63]. The p53 protein initiates the transcription of the effectors of apoptosis and is considered to be the “guardian of the genome,” because it can induce either apoptosis or DNA repair depending on the extent of DNA damage and the efficacy of repair [100]. 

After DNA damage, the increased levels of p53 protein cause the transcriptional activation of p21^WAF1^, thus resulting in cell-cycle arrest [100]. Subsequently, proapoptotic signals converge on the mitochondria, which respond by releasing cytochrome C [104] to activate the final effectors of apoptosis, the caspases [63, 100]. Regulation is achieved by the bcl-2 family of proteins, which exert control on the release of cytochrome C and the caspases [63, 100]. Bax, Bak, Bid, and Bim are pro-apoptotic, whereas Bcl-2, Bcl-XL, and Bcl-W are antiapoptotic member of this protein family [63, 100]. These proteins dimerize and the resulting ratio of inhibition to activation determines a cell's susceptibility to apoptosis [100]; a higher level of bax expression compared to bcl-2 expression correlates with a better outcome for patients with bladder cancer, and early relapses are more common in patients whose tumors express more bcl-2 than bax (both at the mRNA and protein levels) [105]. In a multivariate analysis, bax immunostaining was found to be a significant predictor of better disease-free survival [106], and bax and CD40 ligand are significantly associated with overall survival [107]. Bcl-2 expression has been linked to poorer survival in patients with invasive bladder cancer treated with synchronous chemoradiotherapy [108], and overexpression of bcl-2 has been linked with poorer survival in a group of patients with invasive bladder cancer treated with neoadjuvant cisplatin [109]. 

Fas and CD40 are both members of the TNF-R family of cell-surface proteins [110]. Fas mutations are seen in bladder TCCs, leading to a potential loss of apoptotic function and the generation of circulating soluble forms (sFas) of the protein [110]. *In vitro*, the CD40/CD40 ligand interaction induces apoptosis and growth arrest, as well as inducing the release of proinflammatory cytokines [111], and *in vivo,* TCCs that have invaded the muscle tend to be CD40 negative, in contrast to Ta/T1 TCCs which are mainly CD40 positive [112]. 

These findings demonstrate the interplay among different elements of the apoptotic and cell cycle regulatory pathways in bladder cancer, thus highlighting the importance of p53, Fas, and CD40 as initiators of apoptosis, and while also illustrating the regulation achieved by the balance of pro- and anti-apoptotic proteins.

### 6.4. Replication Potential

The mechanisms discussed above (growth signal autonomy, insensitivity to antigrowth signals, and resistance to apoptosis) lead to the potential for limitless replication [63]. However, these processes alone do not ensure tumor growth; normal cells progress through a limited number of replications before they stop dividing and become senescent [63]. Telomeres are the “counting devices” controlling the number of replications, and comprise hexanucleotide repeats (5′-TTAGGG-3′) that protect the ends of chromosomes [63]. Eventually, after successive replications, the protective telomeres are lost, resulting in chromosomal disarray, crisis, and cell death [63]. However, malignant cells are able to maintain telomere length above a critical threshold to allow unlimited replication. To achieve this, most malignant cells, including bladder cancer cells, upregulate the expression of the enzyme telomerase which adds the hexanucleotide repeats [113]. In bladder cancer, telomerase activity is seen in all grades and stages of bladder TCCs, but not in normal urothelium [114], and this suggests that telomerase activation occurs as an early step in bladder carcinogenesis [115].

### 6.5. Angiogenesis

Proliferative lesions increase their capability for growth and expansion by acquiring angiogenic potential, and this neovascularization is essential for rapid clonal expansion and the development of a macroscopic tumor [63]. Angiogenesis is controlled by the balance of pro- and antiangiogenic signals [63]. Proangiogenic signals include vascular endothelial growth factor (VEGF), FGF1, and FGF2 [116], while thrombospondin-1 is a typical inhibitor of angiogenesis [117]. Thrombospondin-1 is also positively regulated by the p53 protein, so loss of normal p53 function, which occurs in most tumors, can release the inhibition of angiogenesis [117]. 

Streeter and Harris [118] described the role of angiogenesis in bladder cancer in their paper. Bladder TCCs can stimulate more angiogenesis than the normal urothelium, and increased microvessel density is a significant independent prognostic indicator of recurrence and poor survival [119]. In muscle-invasive TCCs, microvessel counts significantly correlate with the presence of occult lymph-node metastases [120]. In addition, high expression of bladder tumor VEGF mRNA is significantly associated with early recurrence, progression to invasion, and high expression of mutantp53 protein [121]; high VEGF serum levels are significantly associated with high stage and grade, vascular invasion, carcinoma in situ, metastases, and worse disease-free survival, [122]. Reduced thrombospondin-1 immunostaining is significantly associated with increased recurrence, decreased survival and the expression of mutant p53 [119], and the *in vitro* secretion of thrombospondin-1 by bladder cancer cells is significantly lower than that secreted by normal urothelial cells [123]. 

The hypoxia-regulated protein, carbonic anhydrase (CA) IX regulates the tissue pH and is a surrogate marker of hypoxia in bladder cancer [124]. CA IX expression also correlates with the VEGF expression in bladder cancer [124]. Significantly more Ta/T1 than invasive bladder cancers strongly express CA IX (82% versus 52%, resp., *P* = 0.03) [125]. However, CA IX expression has no significant correlation with patient survival, suggesting that the dominant role of CA IX occurs early in bladder transitional cell carcinogenesis [124, 125]. 

Angiogenesis and its controlling factors thus have key roles in bladder cancer initiation, progression, and invasion, making this “hallmark” an attractive target for therapeutic manipulation [126].

### 6.6. Invasion and Metastasis

Success in colonizing new sites depends on all of the other five acquired hallmark capabilities discussed, but the initial ability to uncouple from the primary tumor mass depends on the physical characteristics of the cells [63]. The modulation of interactions with neighboring cells and the extracellular matrix and the production of proteases to degrade the extracellular matrix and basement membrane are key processes in adopting an invasive and metastatic phenotype [63]. 

Cadherins are the main mediators of cell-cell adhesion in epithelial tissues, being major components of both the adherens junction and desmosomes [127]. Adhesion is achieved by homodimeric interactions between the extracellular domains of classical cadherins (E-, P-, and N-cadherin) on neighboring cells to form a “zipper”-like structure [128]. Catenins (*α*-, *β*-, and *γ*-catenin and p120) anchor the cadherins to the cell cytoskeleton [128]. E-cadherin (epithelial-cadherin, L-CAM, uvomorulin, and cadherin-1) is a tumor suppressor, and its expression is universally downregulated during epithelial carcinogenesis. P-cadherin (placenta l-cadherin, cadherin-3) behaves in a similar fashion to E-cadherin *in vitro* [129]. N-cadherin (neuronal-cadherin, cadherin-2) has been shown to confer a more malignant phenotype in some tumor model systems [130]. Therefore, it was suggested that cadherin/catenin biology represents a ubiquitous mechanism for epithelial cancer progression [131]. 

In the urinary tract, E-cadherin and *β*-catenin are expressed by membranes throughout the normal urothelium. In bladder TCC, E-cadherin and *β*-catenin expression decrease significantly as the grade and stage increase, and both have been shown to be independent prognostic factors [132]. Mutations and hypermethylation of the E-cadherin gene may play a role in the reduced expression seen in some bladder tumors [133]. However, *β*-catenin-regulated gene transcription does not appear to be important in bladder transitional cell carcinogenesis [134]. P-cadherin is expressed in the membrane only in the basal and parabasal layers of normal urothelium [135]. However, P-cadherin expression increases significantly as the grade and stage progress, and this is associated with a significantly worse survival, with P-cadherin being identified as an independent prognostic factor [136, 137]. 

Desmosomes are also important for maintaining cell-cell adhesion in epithelial tissues; desmosomal adhesion inhibits invasive behavior, suggesting that they have a role in suppressing tumor spread [138]. Invasive TCCs show lower desmosomal density than noninvasive TCCs [139], and modulation of desmosomal proteins appears to be an early event in cell dissociation and the epithelial-mesenchymal transition [140]. However, E-cadherin plays a stronger role than the desmosomal cadherins in the control of invasion of bladder cancer cells *in vitro* [141]. 

CD44 is a ubiquitous cell-surface adhesion molecule involved in cell-cell and cell-matrix interactions, and at least 20 isoforms are known [142]. Quantitative and qualitative changes in CD44 variants have been reported in bladder cancer [143], and some may have prognostic value [144]. 

Matrix-degrading proteases are associated with the cell-surface and facilitate the invasion of cancer cells through epithelial cell layers and into nearby stroma and blood vessels [63]. The 24 human matrix metalloproteinases (MMPs) are a family of proteolytic enzymes that degrade all of the main components of the extracellular matrix and basement membrane, and they are generally overexpressed in human tumors [145]. In bladder cancer, both the expressions of MMP-2 and MMP-9 expression increase significantly as the tumor grade and stage increase, with the MMP-2 expression increasing significantly as the grade and stage increase [146]. *In vitro*, MMP-1 production by bladder cancer cell lines can be stimulated by EGF [146]; FGF-2 has also been shown to have an important regulatory role [83]. There is currently much interest in the clinical use of MMP inhibitors although early trials have been disappointing [83].

### 6.7. Other Factors

Genomic instability allows clones of premalignant cells to reach these six biological endpoints and eventually develop into a tumor. Genomic maintenance systems must be abrogated in order for all of these processes to occur [63]. The roles of the p53 protein and apoptosis are the most prominent of these maintenance systems [63]. The specific chromosomal alterations underlying bladder carcinogenesis have been described [147]. In brief, deletions of chromosome 9 occur in over half of bladder tumors of all grades and stages (9p, 51%; 9q, 57%) [148]. A loss of heterozygosity also occurs on 17p (32%), 11p (32%), 8p (23%), 4p (22%), and 13q (15%), and loss of heterozygosity of 5p, 8p, and 21q are significantly associated with a worse grade and stage [148]. Genomic copy number alterations are also frequent in bladder TCC, with the most frequent changes involving complete or partial loss of 4q (83%) and gain of 20q (78%) [149]. Other frequent losses are of 18q (65%), 8p (65%), 2q (61%), 6q (61%), 3p (56%), 13q (56%), 4p (52%), 6p (52%), 10p (52%), 10q (52%), and 5p (43%) [149]. Many of these loci are currently under further investigation [6, 150, 151]. 

In addition to the mutations of genes, distinct structures and functions of individual cells are achieved by different uses of the same genes and the same sequences with the genome; epigenetic regulation is the mechanism by which gene function is selectively activated or inactivated in cells [152]. The concept of epigenetics has been described as “heritable changes in gene expression that occur without a change in DNA sequence” [152]. The regulation of DNA methylation, histone acetylation, chromatin and chromosomes, transcriptional control, and genome dynamics forms a closely interrelated epigenetic control system [152]. The molecular components of this system have recently been identified and include DNA methyltransferases, methyl-CpG binding proteins, histone-modifying enzymes, chromatin remodeling factors, transcription factors and their regulators (e.g., myc protein/p62, max), and chromosomal proteins, forming an integrated pathway [152]. Epigenetic changes in bladder cancer have been reported, and the alterations are attractive targets for cancer treatment with modulators that demethylate DNA and inhibit histone deacetylases leading to the reactivation of silenced genes [153]. Recently, Vallot et al. [154] have identified a multiple regional epigenetic silencing phenotype characterized by the concomitant epigenetic silencing of several chromosomal regions, which, in bladder cancer, is specifically associated with the carcinoma *in situ* gene expression signature.

MicroRNAs (miRNA) are noncoding RNAs that posttranscriptionally regulate gene expression. Their altered expression and function have been observed in most cancers, including bladder cancer [155]. More than 40 miRNAs have been implicated in urological cancer and many target common carcinogenic pathways. In particular, avoidance of apoptosis, cell proliferation, the epithelial-to-mesenchymal transition, angiogenic signaling, and the generation of androgen independence are targeted or facilitated by more than one miRNA [156]. However, little work has so far been done to evaluate the translational applications of this knowledge to date. However, novel therapeutic strategies have been developed and are under investigation to selectively modulate miRNAs. Such work could therefore potentially make it possible to perform personalized tumor therapy, while also establishing effective disease biomarkers.

## 7. Prevention of Bladder Cancer

For a variety of reasons, bladder cancer is a disease very well suited to chemoprevention. The natural history of bladder cancer is characterized by frequent recurrences, which need to be minimized and carefully monitored. Second, in addition to the genetic susceptibility, environmental factors such as cigarette smoking and other carcinogens, which come in contact with the urothelium, are involved in the pathogenesis of bladder cancer. Therefore, the rationale behind chemoprevention of bladder cancer lies in reducing and/or preventing the contact of these carcinogenic chemicals with the urothelium. Potential chemopreventive compounds administered systemically and excreted in the urine should therefore have the favorable pharmacokinetic property of remaining in close prolonged contact with the urothelium. Diagnostic methods for bladder cancer, which allow easy bladder access and tissue sampling, can be used to evaluate the efficacy of prevention strategies. 

 Three major types of prevention have been defined: primary prevention, which focuses on avoiding the development of cancer in healthy subjects; secondary prevention, which targets premalignant lesions with the intent of avoiding their progression to cancer; tertiary prevention, which focuses on preventing cancer progression in patients diagnosed with early cancer and treated for the disease. Primary and tertiary prevention strategies apply well to bladder cancer. However, primary prevention implies that the trade-off between the risk/inconvenience of intervention and the anticipated benefit is substantial because it pertains to a nonafflicted population. It also implies that a population at risk, in which intervention is warranted, can be identified. These restrictions make primary intervention, albeit attractive conceptually, somewhat difficult to implement in practice. In contrast, tertiary intervention is already widely practiced in bladder cancer in the form of intravesical treatment, but other alternatives with lower toxicity have yet to be fully explored. 

### 7.1. Chemoprevention of Bladder Cancer by Nutritional Factors Green Tea and Catechins

Green tea and its derivatives have been widely investigated as chemopreventive agents for many cancers [157–159]. Epidemiological evidence supports an inverse relationship between increased green tea consumption and bladder cancer risk [160]. The prevalence of bladder cancer appears to be significantly lower among East Asian populations [6], which have much higher rates of tea consumption than in western countries. Bushman reported an approximately twofold increase in the risk of bladder cancer among Japanese families two generations after immigrating to the United States, thus implying that environmental influences including reduced green tea consumption could be contributing factors [161]. Green tea extract contains polyphenols, which have strong antioxidant properties. A transient elevation of the plasma antioxidant activity has been demonstrated with green tea intake, but no proven inhibition of carcinogenesis has yet been clearly established [162]. 

Catechins (epicatechin, epicatechin-3-gallate, epigallocatechin, and epigallocatechin-3-gallate (EGCG)) are thought to play a major role in the anticarcinogenic action of polyphenolic mixtures. These agents inhibit nitrosamine formation and decrease chromosomal damage [163]. Data from several experimental *in vitro* and *in vivo* animal studies support the anti-carcinogenic activity of green tea and its derivatives in bladder cancer models. Kemberling et al. [164] reported the inhibition of the growth of AY-27 rat urothelial cancer cells by intravesical installation of the green tea derivative EGCG. Sato and Matsushima [165] demonstrated the prevention of *N*-butyl-*N*-(4-hydroxybutyl)-nitrosamine (OH-BBN) experimentally induced urinary bladder tumors by green tea leaves, when given before the administration of the carcinogen. Polyphenols can also act as tumor suppressors, blocking the enzyme ornithine decarboxylase (ODC). This enzyme is expressed in bladder tumors, and its action is regulated by EGF [166]. Further evidence comes from a report by Lu et al. [167] demonstrating the promising action of a green tea mixture modulating actin remodeling through Rho activity in *in vitro* human bladder cancer models generated using two nontransformed urothelial cell lines, HUC-PC and MC-T11. 

Green tea supplements were submitted for future investigation and development as tumor preventive agents by the National Cancer Institute [168]. A group of investigators at the University of California, Los Angeles, has initiated an NCI-funded Phase III multicenter clinical trial of green tea supplements. This study is designed as a 3-arm trial comparing green tea polyphenols with the epidermal growth factor receptor antagonist, erlotinib, and placebo in former smokers with intermediate and high-risk nonmuscle invasive bladder cancer in combination with maintenance bacille Calmette-Guérin (BCG). The study requires 110 patients in each of the three arms, and it is currently being conducted at UCLA and the Mayo Clinics in Scottsdale and Rochester.

#### 7.1.1. Fruits and Vegetables

Epidemiological data regarding fruits and vegetables as potential chemopreventive supplements for bladder cancer have been inconsistent and controversial. In 1997, an international review panel considered vegetables as chemopreventive agents for bladder cancer [169]. Increased detoxification of urothelial carcinogens is thought to be the mechanism underlying the protective action of these supplements. In the prospective health professionals follow-up study, an inverse but not statistically significant link between total fruit and vegetable intake and the bladder cancer risk was seen [170]. A high consumption of cruciferous vegetables has been shown to be associated with a significantly reduced risk of bladder cancer compared to a low intake, especially in nonsmokers [170]. Another, large prospective study from Japan concluded that in 39,000 survivors of an atomic bomb attack (15,000 males and 24,000 females), a 50% reduction in bladder cancer risk was associated with high fruit and vegetable intake [171]. The greater effect was linked to the intake of green and yellow vegetables. Overall, the regular consumption of vegetables might have a stronger protective effect against bladder cancer than fruit intake, with a greater impact on the nonsmoking population [172].

#### 7.1.2. Soy Products

Increased intake of soy products has been linked to reduced risk of breast, colon, and prostate cancer [173, 174]. In a recent meta-analysis, Yan and Spitznagel [175] reported an approximately 30% reduction of prostate cancer risk associated with increased soy product consumption. Soy products contain high concentrations of several isoflavones. *In vitro* studies suggest that the protective action of soy may derive in part from induction of G2-M cell cycle arrest, apoptosis, and inhibition of angiogenesis [176]. However, the relationship between soy consumption and bladder cancer risk is still unclear. Surprisingly, a recently reported prospective cohort study of Chinese subjects from Shanghai found a 2.18-fold increase in the risk of bladder cancer associated with high ingestion of soy products after adjustment for age, cigarette smoking, and level of education [177]. This effect was similar in smokers and non-smokers. The conflicting role of soy products in the prevention of prostate and bladder cancer magnifies the importance of further investigation, before any definite recommendations regarding these supplements as chemopreventive agents can be made.

 The isoflavone genistein, a natural soy product, has receptor tyrosine kinase inhibiting activity, as well as phytoestrogenic and other purportedly anticarcinogenic effects [173, 178, 179]. This dietary agent is relatively nontoxic when evaluated in human clinical trials. In *in vitro* systems, genistein has been demonstrated to have antiurothelial cancer activity. Growth inhibition of bladder cancer cells *in vitro* has been associated with inhibition of cyclin B expression, the development of a G2-M cell cycle arrest, and induction of apoptosis [180]. The expression of the EGF-R, whose quantity and distribution are associated with a pan-urothelial abnormalities [181], increases with increasing tumor stage and aggressiveness [182]. The inhibition of EGF-R activity and EGF-mediated responses such as proliferation and cell motility has been reported for genistein in bladder cancer [183]. Additionally, in nonurothelial systems, the expression and activity of cyclooxygenase (COX)-2, an inducible enzyme whose upregulation has been associated with transitional cell carcinogenesis, was downregulated by genistein [184]. In addition, in other cell types critical for tumor growth, such as endothelial cells, VEGF-mediated induction of cyclooxygenase (COX)-2 was inhibited by genistein [185]. Importantly, many markers of these molecular processes can be assessed by immunohistochemistry on formalin fixed, paraffin embedded tissues, and/or bladder washes. While there is not any direct evidence demonstrating genistein's inhibition of EGF-R tyrosine phosphorylation in bladder cancer, genistein is known to inhibit EGF/EGF-R-mediated functions of human bladder cancer cells [183], such as proliferation, invasion, and motility, and it is believed that virtually all functions of the EGF-R are mediated by signaling initiated by EGF-R tyrosine phosphorylation. It is possible that in the case of the human bladder cancer EGF-R, genistein's inhibitory effects are due more to alterations of the downstream signaling pathways rather than EGF-R tyrosine phosphorylation.

#### 7.1.3. Selenium

The role of selenium as a chemopreventive agent for bladder cancer also remains controversial. In the Nurses Health Study with over 121,000 participants, 28 cases of urinary tract cancer were detected [186]. No significant difference was observed in the selenium levels in toenail clippings between cases and controls. Helzlsouer et al. [187] analyzed various serum nutrients from 25,802 subjects in Washington County, Md, USA. During the 12-year period, 35 cases of bladder cancer were identified. The level of selenium was lower in individuals with bladder cancer compared to two matched controls per case. Decreased levels of selenium in the serum were associated with an approximate linear increase in the risk of bladder cancer. However, in a recent nested case-control study of 338 cases and 341 matched controls, prediagnostic selenium levels in archived toe nails were inversely linked with bladder cancer risk in females (*P* for trend = 0.02), but not in males. Further investigation is therefore needed to determine the true role of selenium in the prevention of bladder cancer and whether this role differs by gender.

#### 7.1.4. Garlic

Among the many purported actions of garlic, inhibition of cancer growth is probably the most remarkable. The growth inhibition of various tumor cell lines by ingredients in dietary garlic, such as *S*-allylmercaptocysteine has been reported [188]. Garlic may prevent the suppression of the immune response and thus may decrease the risk of malignancy [189]. Garlic has been evaluated in several epidemiological studies for the prevention of prostate [190], breast [191], colorectal [192], lung [193], and stomach cancer [194] with conflicting results. Further clinical trials are required to understand the true impact of garlic on the reduction of cancer risk.

#### 7.1.5. Fat Consumption

In a multicenter Spanish study, high intake of saturated fat was associated with a greater than two-fold increase in the incidence of bladder cancer [195]. In a meta-analysis of six dietary variables, Steinmaus et al. [196] identified an association between a decreased bladder cancer risk and lower dietary intake of fat. A hospital-based case-control study assessed possible relationships between different dietary supplements and the risk of bladder cancer in Serbia [197]. The cohort was comprised of 130 newly diagnosed bladder cancer patients, and controls were matched by sex, age, and other demographic variables. The authors observed a potentially important role for dietary fat in bladder carcinogenesis (OR = 2.99, 95% CI = 1.16–7.72). There is additional evidence suggesting that this carcinogenic action is dosedependent [198]. If confirmed in larger prospective trials in other populations, a low daily dietary intake of fat could be recommended as one of the possible means to prevent the development of bladder cancer.

#### 7.1.6. Vitamins

Vitamin A, also known as retinol, exists in natural and synthetic forms and can be derived from carotenoids. An important role for vitamin A in the protection and support of epithelial integrity and cell differentiation was established by several experimental studies [199]. Sporn et al. [200] reported inhibition of experimentally-induced transitional and squamous cell bladder carcinomas with 13-*cis*-retinoic acid. It was suggested that vitamin A acts mainly through retinoic acid [201]. Significantly decreased serum levels of retinoids and carotene were found in bladder cancer patients compared to controls in several epidemiological studies [202]. The chemopreventive role of vitamin A may be based on the antioxidant activity of carotenoids via their reduction of DNA damage induced by free radicals [203]. 

A high intake of retinoids can be toxic though and can cause what is known as retinoid syndrome. The manifestations of this syndrome include a steady fever, hypotension, respiratory dysfunction, conjunctivitis, cheilosis, and arthralgia and can even be fatal. A study reported by the National Bladder Cancer Collaborative Group comparing the effect of 13-*cis*-retinoic acid to placebo in patients with rapidly recurring bladder cancer was terminated due to excessive toxicity without any clear-cut benefits in the retinoid arm [204]. 

These potentially harmful effects of vitamin A supplements in some individuals (especially smokers) have motivated investigators to develop synthetic retinoids and retinamides with significantly reduced systemic toxicity and increased activity. Clifford et al. [205] reported *N*-4-hydroxyphenylretinamide (4-HPR or fenretinide) to be an active inducer of bladder cancer cell apoptosis. In a prospective, randomized, double-blind, multicenter trial. Studer et al. [206] assessed the effectiveness of etretinate in preventing recurrence among patients who suffered from superficial bladder cancer. There were no differences in the time to first recurrence between those receiving etretinate and those receiving placebo. However, the interval to second recurrences was significantly longer in the etretinate-treated group (20 versus 12.7 months, resp., *P* = 0.006). This delayed effect of synthetic retinoids may reflect a cumulative drug exposure effect and support continuous treatment, within the limits of acceptable toxicity. However, significant cardiac toxicity occurred in the etretinate arm in this study, and further trials with this agent have not been performed. 

Another multicenter phase III randomized chemoprevention trial compared fenretinide to placebo in patients with TaG1 and G2 or patients with intermediate risk tumors treated with BCG and also found no difference in the time to first recurrence between the two arms, suggesting no benefit for this synthetic vitamin A analog [207, 208]. Furthermore, a 7-year prospective trial of 28,000 male smokers in Finland, no protection against bladder cancer formation was seen for *β*-carotene, alone or with a vitamin E analog. While the findings of preclinical studies may have been promising, to date, no consistent benefit has been seen in the limited clinical trial data available.

Vitamin B6 (pyridoxine) has also been studied as a potential chemopreventive agent for bladder cancer. The proposed mechanism of action of pyridoxine is based on its ability to eliminate the carcinogenic action of some products with an abnormal tryptophan metabolism (kynurenine, 3-hydroxykynurenine, and 3-hydroxyanthranilic acid). These metabolites were found in excessive concentrations in the urine of bladder cancer patients [172]. In a Veterans Administration multicenter study reported by Byar and Blackard [209], pyridoxine was significantly more likely to prevent a nonmuscle invasive bladder cancer than was a placebo, and it was also as effective as thiotepa. Conflicting results have been observed in a double-blind randomized phase III study conducted by the EORTC [172]. A total of 291 patients with non-muscle invasive bladder cancer were randomized 7–14 days after transurethral resection of newly diagnosed bladder tumors to receive 20 mg pyridoxine as a chemopreventive agent or placebo. No difference in the rate or time to tumor recurrence was found. Vitamin B6 may therefore have a mild chemopreventive effect against bladder cancer, but further research studies with a longer followup are needed to provide definitive conclusions.

Vitamin C (ascorbic acid) is a potent water-soluble antioxidant and acts as a free radical scavenger, reducing the formation of bladder carcinogens such as 3-hydro-xanthranilic acid and nitrosamines. Because of these properties, vitamin C can decrease chromosomal damage and eliminate carcinogenic changes in the bladder urothelium [210]. In several human studies, increased intake of vitamin C supplements was associated with a reduction of bladder cancer risk [211]. The health professionals follow-up study found a strong inverse relationship between vitamin C intake and bladder cancer risk in ex-smokers and non-smokers but failed to show the same link with current smokers [212]. There may be a dose limit, beyond which vitamin C may act as an inducer of carcinogenic activity, and high doses should be avoided, especially in patients with a history of bladder cancer [213]. Vitamin C supplements in doses beyond the recommended dietary thresholds may also contribute to hyperoxaluria and an increased risk of oxalate stone formation in individuals predisposed to urinary calculi, thereby potentially limiting its use as a chemopreventive agent [214]. Moreover, the bladder cancer protective effects of vitamin C have not yet been prospectively assessed in placebo-controlled trials.

Vitamin E is a lipid-soluble antioxidant that may act as a chemopreventive agent in several ways. It is an active free radical scavenger and reduces *N*-nitroso compounds attenuating their carcinogenic effect on the urothelium. Additionally, vitamin E modulates the immune function and induces apoptosis [215]. Several studies have reported an inverse relationship between vitamin E intake and bladder cancer risk [212]. The health professional follow-up study showed a 30% reduction in bladder cancer risk in ex- or non-smokers when vitamin E supplements were consumed for more than 10 years. This effect was not found among current smokers [212]. A large epidemiological study reported by Jacobs et al. [216] found decreased bladder cancer mortality in patients who used vitamin E supplements for 10 years or more. However, in a Finish male smoker study that included 28,000 subjects, no protection against bladder cancer development was found with *α*-tocopherol either alone or with the vitamin A analog, *β*-carotene [217]. Prospective trials are required in order to determine the long-term safety and role of vitamin E as a chemopreventive agent. It should be remembered that in high doses, vitamin E may increase the bladder cancer risk, and overdoses can be fatal [218, 219].

#### 7.1.7. Lycopenes

Lycopenes are unsaturated, nonprovitamin A carotenoids found in tomatoes, guava, rose hip, watermelon, and pink grapefruit, giving these fruits their red color. Lycopenes concentrate in specific compartments of the body, including the breast, prostate, and pancreas. Like other carotenoids, they are potent antioxidants and have been suggested to reduce the risk of bladder cancer. A study of the impact of a 12-week treatment with tomato juice in rats initiated with a bladder carcinogen, OH-BBN, showed a decrease in tumor number without affecting the tumor incidence [220].

#### 7.1.8. Linoleic Acid

Linoleic acid is a polyunsaturated fatty acid found in vegetable oils and meats. Both linoleic acid and its stereoisomer, conjugated linoleic acid, which is derived from ruminant animals and their dairy products, have demonstrated an ability to suppress proliferation and enhance apoptosis in bladder cancer cell lines [221, 222]. While linoleic acid exerted no effects on the growth of cancer cells, conjugated linoleic acid inhibited the growth in a dose-dependent manner [221]. Although these recent *in vitro* data are encouraging, *in vivo* studies are necessary before linoleic acid and conjugated linoleic acid can be advocated to prevent bladder cancer development.

#### 7.1.9. Other Nonnutritional Agents

Based on the findings that antioxidative substances modulate bladder carcinogenesis in rodents [223], our group reported several natural compounds that effectively suppressed experimental bladder carcinogenesis in rats and mice. They include astaxanthin [224], protocatechuic acid [225], diosmin [226], hesperidin [226], silymarin [227], 1,4-phenylene diisothiocyanate [228], and *β*-cryptoxanthin [229]. These compounds are ready to be evaluated for efficacy in clinical trials.

### 7.2. Chemoprevention of Bladder Cancer by Synthetic Chemicals (Drugs) Difluoromethylornithine (DFMO)

In the induction of the synthesis of polyamines through the activation of the enzyme, ODC has been reported to be closely associated with tumor promotion and the activities of both hormones and growth-promoting factors [73]. Increased ODC activity has been found in a variety of cancers, including urothelial cancer [73], and its activity can be blocked by using a noncompetitive inhibitor, DFMO. DFMO has been found to selectively inhibit the growth of malignant human urothelial cells *in vitro* compared to normal cells. This effect can be reversed by supplementing the medium with the polyamines putrescine or spermidine, important in DNA synthesis and gene expression [73], indicating that its primary inhibiting effect is likely mediated through polyamine depletion. DFMO also inhibits the development of carcinogen-induced urothelial cancer in rodents and other models [73, 230]. This agent has been tested in elderly patients (including those with bladder cancer) in oral doses that are well tolerated [231] and that reduce ODC activities in malignant urothelial tissue [232]. 

A study by Messing et al. [233] was undertaken to determine if DFMO could prevent recurrences of completely resected low-risk bladder cancer compared with placebo and to determine the incidence and severity of adverse events associated with receiving DFMO for up to 1 year in this patient population. A randomized, double blind, placebo-controlled trial was conducted in patients with grade 1 or 2, stage Ta or TI, newly diagnosed or occasionally recurrent (less than one recurrence per year and no more than three total recurrences) urothelial cancers. Within 12 weeks of complete endoscopic resection, patients were randomized and started to receive 1 g of oral DFMO or placebo once per day, with treatment to be continued for 12 months or until histologically confirmed tumor recurrence, whichever was sooner. In the largest bladder cancer prevention trial ever conducted, 454 patients were randomized at 70 clinical sites in the United States, Canada, and the United Kingdom. A total of 194 patients (90 DFMO and 104 placebo) completed the 12-month treatment period, while 248 (130 DFMO, 118 placebo) discontinued treatment early. Toxicity was low, with adverse events noted in 6.6% of patients receiving DFMO and 5.7% on placebo. However, DFMO did not reduce the frequency of recurrence or delay the time to first or subsequent recurrences in the population as a whole, nor in any stratification category. Forty-four percent of recurrences in the DFMO arm and 51% in the placebo arm occurred within 6 months of randomization, and another 29% in the DFMO arm and 28% in the placebo arm occurred within the next 6 months. Over 73% of first recurrences in both groups occurred within the first year of the study.

#### 7.2.1. Nonsteroidal Anti-Inflammatory Drugs (NSAIDs) and Selective COX-2 Inhibitors

NSAIDs are one of the most potent bladder cancer chemopreventive agents in preclinical studies [234]. This class of drugs blocks the expression of COX, the key enzyme in prostaglandin synthesis. COX exists in two predominant isoforms, COX-1 and COX-2. COX-1 is ubiquitously expressed and constitutively active in the gastric mucosa, kidneys, and platelets (among other cell types). Inhibition of COX-1 by nonselective NSAIDs leads to platelet dysfunction, gastritis, and occasional renal dysfunction, and therefore these agents are not always well tolerated during chronic administration. In a large prospective study of aspirin users, no relationship was found between aspirin intake and urothelial cancer risk [235]. However, a recent case-controlled study from California demonstrated a 20% decrease in bladder cancer risk among the population with regular use of NSAIDs [236]. The chemopreventive effect of NSAIDs is thought to result from COX-2 inhibition. COX-2 is inducible by inflammatory cytokines, hormones, growth factors and tumor promoters. Prostaglandin E_2_ (PGE_2_) is the end product of eicosanoid synthesis which is mediated by COX-1 and COX-2. PGE_2_ can stimulate cell proliferation and motility while inhibiting apoptosis and immune surveillance. Similarly, PGE_2_ can stimulate tumor-driven angiogenesis. Inhibition of COX-2 may block these and other procarcinogenic properties, thus contributing to the chemopreventive activities of this class of selective NSAIDs [237]. 

Data from preclinical *in vivo* studies supports the link between the up-regulation of COX-2 and the occurrence of bladder cancer [238]. The tumor suppressive effect of celecoxib, a selective COX-2 inhibitor, is thought to be dose-dependent, and stronger at higher doses [239]. Gee et al. [240] evaluated the chemopreventive and therapeutic properties of the selective COX-2 inhibitors, celecoxib, and NS-398, on three different human bladder cancer cell lines (UM-UC-1, 3, and 6). Celecoxib induced significant apoptosis in all three cell lines, acting through the downregulation of Bcl-2. Both NS-398 and celecoxib demonstrated dose-dependent tumor cell growth inhibition modulated by a significant reduction in the number of cells in the S-phase [240]. The growth inhibitory activity of these selective COX-2 inhibitors was independent of the COX-2 expression levels, thus suggesting the presence of additional anticarcinogenic effects, other than inhibition of COX-2. 

In human bladder tissue, COX-2 is expressed in urothelial dysplasia, Tis, and in the majority of TCCs, independent of stage and grade [241, 242]. Shariat et al. [243] described the patterns of COX-2 expression in patients with carcinoma in situ and/or stage T1 TCC and reported an association of >10% immunoreactivity with the progression of Tis but not T1 disease, and no association with overall survival. These clinical findings suggest that selective COX-2 inhibitors are an appropriate class of drugs to evaluate in human clinical prevention trials because they have a higher tolerability profile than nonselective NSAIDs, which predominantly block COX-1 [156, 231]. A multicenter study led by MD Anderson Cancer Center and supported by the National Cancer Institute and Pfizer Incorporated to evaluate a selective COX-2 inhibitor in patients with nonmuscle invasive bladder cancer treated with BCG is ongoing [244]. Patients who showed a complete response to an induction course of 6 instillations of BCG and completed at least one 3-week maintenance course of BCG were eligible for randomization to 1 or 2 years of 200 mg twice per day of celecoxib or a placebo. The primary endpoint of the study is the time to recurrence after 12 months of treatment with the study drug. The study design called for 156 evaluable patients in each arm with a power of 80% to detect a 41% reduction in recurrence. Secondary aims include assessment of the modulation of several biomarkers, including prostaglandin dehydrogenase (PGDH), basic fibroblast growth factor, the autocrine motility factor receptor, and markers of proliferation and apoptosis. Modulation of these biomarkers will be examined for a correlation with tumor recurrence. Accrual has been completed, but not all patients have completed the treatment protocol, and the results are thus pending. 

It should be remembered that long-term exposure to COX-2 inhibitors has the potential to be harmful and has been associated with a well-publicized small, but significant, elevation in cardiovascular (CV) events [245]. There has been no significant CV toxicity in a celecoxib Trial, but several selective NSAIDs have been taken off the market, and the ability to test this class of agents in future bladder cancer prevention trials may be limited. This is particularly true for primary prevention, which is performed in subjects who have never had bladder cancer and are not considered to be at as high of a risk as those who have already had tumors.

#### 7.2.2. Statins

A class of hydroxymethylglutaryl-coenzyme A reductase inhibitors, the statins, has been suggested to decrease the risk of multiple cancers, including bladder cancer and prostate cancer. The mechanism(s) responsible for this effect is unclear although cholesterol is considered to be one of the mediators of tumorigenesis. It is therefore logical that drugs that decrease lipoproteins, such as the statins, can decrease cancer growth and tumor progression [246]. *In vivo* studies of statins are necessary before clinical trials are planned.

#### 7.2.3. Antibiotics

Oltipraz, an antiparasitic agent (5-[2-pyrazinyl]-4-methyl-1,2-3-thione), originally developed as an antischistosomal medication, was found to protect against chemically induced carcinogens in the lung, stomach, colon, and urinary bladder in animals. The mechanisms of oltipraz action include enhancement of DNA repair processes, induction of phase I enzymes (cytochrome P450) that enhance carcinogen detoxification, and nucleophilic trapping of reactive intermediates, among others. Oltipraz inhibits the carcinogenesis induced by polycyclic aromatic hydrocarbons and *N*-nitrosamine agents that constitute some of the carcinogenic components of tobacco. Recent reports have indicated that oltipraz may have anticarcinogenic activity as well. 

The antiangiogenic and antitumor efficacy of oltipraz in nude mice was evaluated by measuring its effects on neovascularization in subcutaneous implants seeded with vascular endothelial growth factor and basic fibroblast growth factor-stimulated porcine aortic endothelial cells, and on tumor growth and angiogenesis in SVR murine angiosarcoma xenografts implanted subcutaneously [247]. A dose-dependent reduction (0.4–100 *μ*M) in microvessel genesis was observed in bioassays after oltipraz treatment, with decrease of 100% in the rat aortic ring assay at the highest concentration (*P* < 0.01). Administration of the oltipraz to athymic mice bearing established subcutaneous SVR angiosarcoma xenografts for 10 days resulted in a significant inhibition of tumor growth as early as day 4 after the beginning of treatment (*P* < 0.005), with a maximum inhibition of tumor growth (81%, *P* < 0.001) relative to vehicle-treated mice by day 10.

The efficacy of oltipraz approached the angiogenesis inhibiting effects of known anti-angiogenic agents, such as SU 5416 (semaxanib). Phase I trials conducted in the United States have shown that the maximum tolerated dose of oltipraz is approximately 125 mg/day, over a 6-month period [248]. Dose-limiting toxicities included photosensitivity, heat intolerance, gastrointestinal toxicities, and neurologic toxicities [249, 250]. Oltipraz is unique in its multiple mechanisms of action as an antischistosomal, antiangiogenic and anticarcinogenic agent. Its chemopreventive effects should therefore be beneficial for patients with a history of *Schistosoma haematobium* bladder infections who are known to be at an increased risk for developing bladder cancer as well as in smokers. This agent may also be effective in the treatment of advanced stage cancers. However, oltipraz has not yet been studied in phase III chemopreventive trials.

## 8. Animal Models of Bladder Cancer

Appropriate and valid animal models for urinary bladder carcinogenesis must be similar to human bladder cancer in their histology, biochemical properties, molecular and genetic characteristics, natural history, and biological behavior [251]. A simple and natural method for the administration of the carcinogen is required; the carcinogen ought to be nontoxic and should ideally affect only the urothelium. 

Three chemicals have proven to be particularly effective to induce bladder neoplasms. These chemicals include *N*-[4-(5-nitro-2-furyl)-2-thiazolyl] formamide (FANFT), OH-BBN, and *N*-methyl-*N*-nitrosourea (MNU) [252]. These compounds are complete carcinogens, and the total dose has a greater effect when administered as several fractions, that is, the effect of the fractions is synergistic rather than additive. The grade of cellular atypia and the extent of invasion increase as the dose of carcinogen increases, as well as when the experimental period is extended [253]. A fried food mutagen 2-amino-3-methylimidazo[4,5-*f*]quinoline, also results in bladder cancer [254]. Among them, OH-BBN is the most suitable urinary bladder carcinogen for animal models, since its carcinogenic potential is essentially limited to this organ, and it is probably the most commonly-referenced experimental bladder carcinogen [255]. Bladder tumors induced by OH-BBN in rats and mice resemble their human counterparts [256]. OH-BBN is a metabolite of the symmetric dibutylnitrosamine (DBN) [257]. In rats, both were demonstrated to be urinary bladder carcinogens, with OH-BBN being specific to the urinary bladder, because DBN also induced tumors of the liver, lung, kidney and esophagus [258]. Similar findings have been shown in mice [259]. OH-BBN is bladder specific not only in rats, but also in mice and dogs. A 100% incidence of bladder tumors can be induced in rats and mice by continuous and prolonged administration of OH-BBN in drinking water. The animal bladder carcinogenesis model using OH-BBN, thus, is useful for investigating bladder carcinogenesis, screening cancer chemopreventive agents, and determining therapeutic efficacy of new anticancer agents.

Multistage models of carcinogenesis proposed to explain the patterns of tumor development observed in the urinary bladder involve the initiation of neoplastic changes in a few cells by a threshold dose of carcinogen, followed by conversion of these latent tumor cells into an autonomous cancer by further doses of the same and/other carcinogens, and/or promoting agents. In the urinary bladder of mice and rats, neoplastic changes can be initiated by a few weeks of treatment with low doses of the chemical carcinogens described above. Animals subsequently exposed to promoter compounds, such as sodium and potassium salts, urolithiasis-inducing agents, and certain antioxidants, develop increased number of bladder cancer [213, 260].

## 9. Conclusion

We now have a good understanding of the pathobiology of bladder cancer development from the large body of evidence provided by experimental and epidemiological studies. In addition, numerous experimental and epidemiological studies have shown a promising response of bladder cancer to several potential chemopreventive agents. However, limited clinical data in well-designed prospective trials for the prevention of bladder cancer have been less encouraging. Future large randomized clinical trials are needed before definite recommendations can be established regarding efforts to reduce bladder cancer recurrence rates, and to improve overall outcomes. The clinical challenges related to preventing and treating bladder cancer will increase as our population ages and chemoprevention shows promise in this battle, because of the recurrent nature of bladder cancer.

##  Conflict of Interests

The authors declared that they have no conflict of interest.

## Abbreviations

BCG: Bacille Calmette-Guérin
BCPN: *N*-butyl-*N*-(3-carboxybutyl) nitrosamine
BTA: Bladder tumor antigen
CA: Carbonic anhydrase
CDKs: Cyclin-dependent kinases
COX: Cyclooxygenase
CV: Cardiovascular
DBN: Dibutylnitrosamine
DFMO: Difluoromethylornithine
EGCG: Epigallocatechin-3-gallate
EGF: Epidermal growth factor
EGF-R: Epidermal growth factor receptor
FDP: Fibrin degradation product
FGFs: fibroblast growth factors
FGF-R3: Fibroblast growth factor receptor 3
FANFT: *N*-[4-(5-nitro-2-furyl)-2-thiazolyl] formamide
HA: Hyaluronic acid
HAase: Hyaluronidase
HGF: Hepatocyte growth factor
4-HPR: *N*-4-hydroxyphenylretinamide
HPV: Human papillomavirus
IGF: Insulin-like growth factor
MAPK: Mitogen-activated protein kinase
miRNA: MicroRNAs
MMPs: Matrix metalloproteinases
MNU: *N*-methyl-*N*-nitrosurea
NMP: nuclear matrix protein
NSAIDs: Nonsteroidal ant-inflammatory drugs
ODC: Ornithine decarboxylase
OH-BBN: *N*-butyl-*N*-(4-hydroxybutyl)-nitrosamine
PGDH: Prostaglandin dehydrogenase
PGE_2_: Prostaglandin E_2_
TCC: Transitional cell carcinoma
TGF: Transforming growth factor
TNF: Tumor necrosis factor
VEGF: Vascular endothelial growth factor.
